# Contextualizing neuro-collaborations: reflections on a transdisciplinary fMRI lie detection experiment

**DOI:** 10.3389/fnhum.2014.00149

**Published:** 2014-03-31

**Authors:** Melissa M. Littlefield, Des Fitzgerald, Kasper Knudsen, James Tonks, Martin J. Dietz

**Affiliations:** ^1^Department of Kinesiology and Community Health, University of Illinois at Urbana-ChampaignUrbana, IL, USA; ^2^Department of English, University of Illinois at Urbana-ChampaignUrbana, IL, USA; ^3^Department of Social Science, Health and Medicine, Kings College LondonLondon, UK; ^4^Interacting Minds Center, Aarhus UniversityAarhus, Denmark; ^5^Department of Culture and Society, Section for Anthropology and Ethnography, Aarhus UniversityAarhus, Denmark; ^6^Dame Hannah Rogers Trust and Department of Psychology, University of ExeterExeter, UK; ^7^Center for Functionally Integrative Neuroscience, Institute of Clinical Medicine, Aarhus UniversityAarhus, Denmark

**Keywords:** neuro-collaboration, disciplinary double consciousness, transdisciplinarity, fMRI lie detection, critical neuroscience

## Abstract

Recent neuroscience initiatives (including the E.U.’s Human Brain Project and the U.S.’s BRAIN Initiative) have reinvigorated discussions about the possibilities for transdisciplinary collaboration between the neurosciences, the social sciences, and the humanities. As STS scholars have argued for decades, however, such inter- and transdisciplinary collaborations are potentially fraught with tensions between researchers. This essay build on such claims by arguing that the tensions of transdisciplinary research also exist within researchers’ own experiences of working between disciplines - a phenomenon that we call “disciplinary double consciousness” (DDC). Building on previous work that has characterized similar spaces (and especially on the Critical Neuroscience literature), we argue that “neuro-collaborations” inevitably engage researchers in DDC - a phenomenon that allows us to explore the useful dissonance that researchers can experience when working between a “home” discipline and a secondary discipline. Our case study is a five-year research project in functional magnetic resonance imaging (fMRI) lie detection involving a transdisciplinary research team made up of social scientists, a neuroscientist, and a humanist. In addition to theorizing neuro-collaborations from the inside-out, this essay presents practical suggestions for developing transdisciplinary infrastructures that could support future neuro-collaborations.

It is unlikely that disciplinary coherence can be maintained in these new circumstances

“Discipline” (Turner, 2006, 186)

The recent U.S. BRIAN Initiative and the E.U. Human Brain Project (HBP) have been likened to the Human Genome Project in scope and potential for revolutionary change(s) to medicine, neuroscience, and computing.^1^ Amidst the hype is also the real hope that a centralized system for collaboration between neuroscientific disciplines, around specific, large-scale neuroscientific projects, will produce better results in less time. As the HBP “Overview” describes it, “one of the major obstacles to understanding the human brain is the fragmentation of brain research and the data it produces. Our most urgent need is thus a concerted international effort… to integrate this data in a unified picture of the brain as a single multi-level system” (Human Brain Project, 2014a). For the U.S.-based BRAIN Initiative, the focus differs (from building a model of the brain to mapping brain connections), but the sense of collaborative exigency persists, “[recent] discoveries have yielded unprecedented opportunities for integration across scientific fields” (National Institutes of Health, BRAIN Initiative, 2014). The basis for both programs is large-scale, transdisciplinary collaborations. Or, to quote Karlheinz Meier’s contribution to the “What People are Saying” scroll-bar at the bottom of the HBP homepage: “Collaborate, collaborate, collaborate. This is our opportunity” (Human Brain Project, 2014b).

As research in Science and Technology Studies has demonstrated, collaboration is often easier to imagine and/or critique than to enact. Discussions of “Trading Zones” (Galison, 1997; Collins et al., 2007), transdisciplinarity (Nicolescu, 2008; Frazzetto and Anker, 2009; Frazzetto, 2011), and boundary work (Gieryn, 1983, 1996, 1999) have mapped some of the difficulties of working together – particularly when that work requires crossing disciplinary lines. Discussions of interdisciplinarity’s complex history and relation to other (multi-, trans-, and cross-) disciplinary mergers have helped to reveal the multiplicity of research that translates across the social and natural sciences (Barry and Born, 2013). Likewise, numerous inter-/transdisciplinary projects have both tested the limits of collaborative endeavors and engendered novel theorizations of what it means to work between departments and disciplines. In this vein, we might look at collaborations whose continuing mission is increased reflexivity, such as Simon Penny’s work on the Arts Computational Engineering Program at UC Irvine^2^ and research centers such as the Center for Nanotechnology in Society at ASU.^3^ In short, the collaborative happenings with/in the neurosciences are not a novel phenomenon, and, indeed, they recall larger and longer conversations about the fraught intersections between biology-and the social sciences (Benton, 1991; Williams et al., 2003; Meloni, 2013; Rose, 2013). While the neurosciences have always already enacted collaborations between scientific and clinical fields (e.g., biology, chemistry, physics, psychology), an emergent suite of cosmopolitan “neurodisciplines” (centering on neuroscience, but moving into the concerns of the humanities and social sciences, as well as other natural sciences) have reinvigorated discussions concerning transdisciplinarity. These neurodisciplines have also bolstered our capacities for collaborative research models that bring together the neurosciences, humanities, and social sciences (Choudhury and Slaby, 2011; Pickersgill and Van Keulen, 2011; Littlefield and Johnson, 2012; Ortega and Vidal, 2012; Rose and Abi-Rached, 2013).

But if these neuro-conversations-and others-have led to conceptual, historical and bioethical analyses of neuroethics (Racine et al., 2005), neurorhetorics (Pruchnic, 2008 and Jack, 2012), and the proliferation of the neurodisciplines, there is less discussion about the *practical infrastructures* that actually make such neuro-collaboration possible. Beginning that discussion is the goal of this paper. In a related analysis, we have considered some of the affective and political components of actually doing a transdisciplinary neuroscience (Fitzgerald et al., 2014). In this paper, by contrast, we re-focus on the infrastructural pragmatics of neuro-collaboration, and its relationship to neuroscience as such. In what follows, we introduce the term “neuro-collaboration” as shorthand for myriad working groups that attempt to pragmatically splice together the neurosciences, the humanities, and the social sciences. Our purpose here is not to define or circumscribe what these collaborations might look like; instead, we focus on the complications of collaborations that bring various external disciplines into conversation with the neurosciences.^4^One possible rubric for neuro-collaboration that has risen to prominence since its introduction in 2009 is Critical Neuroscience (Choudhury et al., 2009). The nescient field of Critical Neuroscience has proposed direct intervention in neuroscientific studies in order “to push experimental work in alternative directions” (Choudhury and Slaby, 2011, 13). However, and as this special issue implies, there is still a live question about the degree to which such collaborations between the neurosciences and the humanities and/or social sciences may actually produce something new. Do these collaborators bring “critical” interventions to the neurosciences that are/were lacking? Or, is there a real possibility, given the hierarchical structuring of the academy, that neuro-collaborations might just be another term for neuroscience? We raise these questions because it is not always clear what neuro-collaborations look like in an extended application; moreover, we do not yet know what types of tangible results researchers can expect – or even hope for – from these kinds of collaborations. Critical Neuroscience is a helpful starting point; but, thus far, it underestimates - or has not yet accounted for - forms of action, consciousness and subjectivity that are required for working through collaborative domains where, as per the emerging neurodisciplines, neither prestige nor resources nor insights are distributed evenly. And because of such asymmetries, the relationship between “neuro-collaboration” and “neuroscience” is not always clear. That relationship is at the heart of what follows.

In this paper, we contend that extended deployments in other disciplines for the purpose of neuro-collaboration(s) can cause a sense of what we call disciplinary double consciousness (DDC) among research team members (cf. Hurley, 2003, 7). In order to prevent neuro-collaborations-or “Critical Neuroscience,” for that matter-from becoming, simply, neuroscience, research team members may need to learn not only how to recognize the symptoms of DDC, but also to experience DDC as a useful collaborative position and tool. In this context, we contend that DDC can be productive and informative, even as it reveals hierarchical power relations between the neurosciences and its potential humanities and social science collaborators. Our arguments are based on a five year experimental collaboration between a neuroscientist, a humanist, and several social scientists that was originally intended to challenge current functional magnetic resonance imaging (fMRI) lie detection paradigms, but which also turned into a meta-experiment in neuro-collaboration.^5^

## LITERATURE REVIEW: DOUBLE CONSCIOUSNESS WITHIN THE TRADING ZONES

Properly speaking, *a man has as many social selves as there are individuals who recognize him* and carry an image of him in their mind. [... ] From this, there results what practically is a division of the man into several selves; this may be a discordant splitting, as where one is afraid to let one set of his acquaintances know him as he is elsewhere; or it may be a perfectly harmonious division of labor, as where one tender to his children is stern to the soldiers or prisoners under his command.

William James, *Principles of Psychology*, 1890, 294

The term “double consciousness” has a long history, typically associated with the experience of African American racialized identities, becoming particularly salient in the wake of the American civil war (Du Bois, 1994). For W. E. B. Du Bois, “double consciousness” describes how African-American identities are informed by and through the contemptuous eyes of another-the white ruling class; for example. Through his famous image of “the veil,” Du Bois captures precisely the simultaneous feeling of being both inside and outside an identity, a nation, a culture, an historical experience - and the intellectual and affective labors of tacking back and forth between such senses of doubling. Recently, scholars have drawn on Du Bois’s account to describe myriad subcultures in which identity and subjectivity are also suspended in complex and doubled relationships with often-oppressive-but-inescapable larger cultures: female boxers at the turn of the twentieth century (Gammel, 2012), the place of “character” and moral responsibility in criminal law (Lacey, 2010), and the relationship between the ideal and the real in nineteenth century literature (Mills, 2011), just to name a few. This expansive use of double consciousness inspires our application to neuro-collaborations.

Likewise, our use of double consciousness derives in part from the scientific genealogies of the term from which Du Bois may have drawn his theories about the feeling of African-American identity in the United States. As Dickson D. Bruce Jr. (and Arnold Rampersad before him) have articulated, Du Bois’s double consciousness drew from at least two traditions, one figurative and one medical: on the one hand, European Romanticism and American Transcendentalism and, on the other, the then-emerging field of psychology. In his essay, “W. E. B. Du Bois and the Idea of Double Consciousness,” Bruce (1992) contends that “as a medical term “double consciousness” already had a long history by the 1890s, having been the subject of rather extensive experimentation and debate for at least seventy-five years” (303). He explores this history of the term via William James, who was one of Du Bois’s mentors while Du Bois studied at Harvard, and older histories that have roots in the 1817 case of Mary Reynolds, a woman who had at least two distinct consciousness that alternated over a 15 year period in her life.

That “double consciousness” is, itself, a term with a complex (and simultaneous) disciplinary, scientific and political history is relevant to our own discussions. We do not draw analogies between racialized and disciplinary identities. Nonetheless, given the current state of the academy as a micro-world of power and prestige divided along non-natural but still hard lines of disciplinarity, we want to draw on Du Bois’s conceptual framework. DDC illuminates the sense, throughout this experience, of being not quite one thing nor another, of being captured within the powerful categorical gaze of some other rubric, and of learning to make ourselves, and our intellectual identities, as neither quite scientist or non-scientist, as both object and ally of some hardly seen external force. This also jibes well with William James’ theorizations of double or alternating consciousness, to which we will turn in a moment.

We define DDC as the sensation of dissonance experienced (cognitively, affectively, conceptually, or otherwise) by being caught between a “home” discipline and position as a scholar on an extended deployment in some secondary discipline; the position-and the term-are contingent on a series of historically- and socially embedded disparities between disciplinary methods, practices, and theoretical foundations. The more radical the disparity has been made to be, the larger the sense of DDC. Here, we have chosen the term “deployment” to describe the experiences of humanists and social scientists who are working in the neurosciences because it captures the physical transition from one space to another, potentially from one culture to another. The term also captures the quasi-militaristic and action-oriented aspects of the project at hand: to not only strategically inhabit another discipline, but also prompt action that results in transdisciplinary outcomes. We note here that DDC is not limited to neuro-collaborations and, indeed, could be applied to myriad collaborative situations. Due to limitations of space, we focus here on neuro-collaborations specifically, but believe future scholarship should be build outward to other fields and collaborative endeavors.

According to William James’s analysis, one’s sense of double or alternating consciousness has several possible causes, including individual pathology, but also extending to infrastructural systems. As he notes in *The Principles of Psychology*, “we must admit that organized systems of paths can be thrown out of gear with others, so that the processes in one system give rise to one consciousness, and those of another system to another *simultaneously* existing consciousness” (James, 1890, 399). In this brief statement, the implications for disciplinarity become articulable: we can extrapolate that organized systems of paths, including modern disciplines have been “thrown out of gear with others” through the various processes of boundary construction, expertise building, and professionalization. Scholars working in one field are introduced to and indoctrinated in one system or consciousness may find that they not only experience a different consciousness when working between disciplines, but that they experience the multiplicity of consciousness *simultaneously*, a position that is irreducible to one or another epistemology or ontology.

The conception of a simultaneously existing consciousness sheds new light on theorizations of collaboration, particularly notions of trading zones (Galison, 1997; Collins et al., 2007) and, more recently, Critical Neuroscience. First, the notion of trading zones, as it has been rearticulated by Collins et al. (2007) argues that there are distinct relationships between disciplinary players when they work in or simply meet at disciplinary borders. Collins et al. (2007) divide Galison’s original conception of the trading zone into four parts: Inter-Language, Fractures, Subversive, and Enforced (2007, 659). Each represents a differential distribution of power and expertise between fields. If we were to place the recent surge in neuro-collaborations on such a chart, we might locate them somewhere between Fractured and Subversive: not yet a recognized creole like the nanosciences, but not as coercive as the slave/master relation in the Enforced category. However, within the Fractured and Subversive categories-two locations that might describe neuro-collaborations-what is less explicit are the disciplinary consciousnesses of researchers working within such paradigms. Collins et al. (2007) approach this issue by noting that there is a particular-and special-place for the scholar who can move fluidly between fields: “indeed, it is precisely the continuing discontinuity between the cultures that enables *the individual with interactional expertise*, and who thus has a mastery of both languages, to maintain their special role” (Collins et al., 2007, 662, our emphasis). What Collins and his colleagues pay less attention to, however, are the subjective experiences of human beings working across these boundaries, and the role of such experience in boundary-formation; the distinctions they describe, and the communities that make them up, are both enacted and lived as forms of subjectivity and consciousness. Thus we draw attention to the way that consciousness might be an unexplored site for illuminating the traffic between disciplines, as well as the barriers to that traffic. In particular, we explore how shifts in consciousness might both sustain and mediate the very boundaries that Collins and his colleagues throw into relief. If we find the interactional polyglot researcher an enticing figure, we also wonder about the degree to which she may experience a strong sense of DDC – an experience that might well account for her special role in the collaborative team.

Likewise, it is imperative that we recognize the power of DDC to disrupt the transmogrification of a fractured trading zone into a homogeneous one. Collins et al. (2007); note that

Over an even longer time period it may even be the case that, as with biochemistry, a distinct new discipline emerges. [...] This possibility is realized when departments are set up, textbooks are produced and what was once a radical and innovative experiment becomes a normal science research program.... New recruits to the discipline will now find the cultural hegemony so strong that they have no choice but to abandon any ideas they bring with them and accept the dominant culture. Anything strange sucked into its domain will be subverted by the dominant culture of the new science.

(Collins et al., 2007, 663–664)

As we have already mentioned, neuro-collaborations have the potential to turn into something else, including, simply, neuroscience. But our suggestion is that the recognition and productive use of DDC may also make this transition less automatic and more fraught-something that could help maintain the transdisciplinary, or even critical neuroscientific edge of neuro-collaborations. In short, there might be more than hegemony (i.e., simply doing neuroscience) at stake over “the Western horizon.” Maintaining attention to the role of consciousness, and its potential for producing/living with a form of doubling introduces a new way to think about the development of this taxonomy.

Thus, for Critical Neuroscience, which assumes the potential for an inter-language trading zone, if not a creole discipline, the existence or experience of DDC could help to guard against a slippage into neuroscience as such. In the call for transdisciplinary research teams, Critical Neuroscience could incorporate a record of DDC as a useful third position. According to such a model, collaboration implies the recognition, if not creation, of a transdisciplinary space that is irreducible to any one field. Choudhury and Slaby (2011) argue that “practitioners of Critical Neuroscience might temporarily form specific project groups to collaborate for a certain time on specific topics, thereby applying all the relevant tools to trace the trajectory of a given theme or brain “fact” and to plan new experiments where relevant” (Choudhury et al., 2009, 69). If we revise this statement to reflect DDC, we would articulate that this relationship is neither temporary nor is it centrally fixated on the neurosciences; instead, we have come to understand it as an extended deployment intent on moving beyond and outside of the often dominant natural sciences. Indeed, focusing on the formation of disciplinary consciousness reminds us that aggregation is not only at the level of groups of individuals - that aggregation might also be a question of one person’s subjectivity, a function *within* the individual, and not only a product of her interactions. So in this sense, collaboration does not stop at the boundaries of the body - it is an interior process of churn too, irrespective of what Collins et al. (2007) call hegemony.

## CASE STUDY: NEURO-COLLABORATION IN AN fMRI LIE DETECTION EXPERIMENT

Our research group met for the first time at a small hotel in Vienna. The authors were among a small group of early career scholars invited to participate in a new kind of event: a “Neuroschool” sponsored by the European Neuroscience and Society Network (ENSN). The ENSN was funded by the European Science Foundation for five years between 2007–2012, and was initiated and chaired by a team from the BIOS Centre, then affiliated with the London School of Economics. Under their rubric of “mutual exchange” (King’s College London, 2007a) the ENSN sponsored four Neuroschools (Rome 2008, Vienna 2009, Wurzburg 2010, Bergen 2011), which sought theoretically-and practically-to redress the problems of education and training across disciplinary divides. While the school’s name gives notable weight to the “neuro” part of the equation, the ENSN’s larger goal of transdisciplinarity is explicitly locatable within the environs of the Neuroschools: “Intended for early career neuroscientists and social scientists, Neuroschools offer a symmetrically transdisciplinary environment for cross-tutoring and sharing of ideas and for innovative, critical thinking about key issues in modern neuroscience” (King’s College London, 2007b). Here, discourses of mutual exchange stud the description: “symmetrically transdisciplinary environment,” “cross-tutoring,” and the “sharing of ideas.” In short, the ENSN Neuroschools sought to foster research environments that do not simply bring disciplines together (interdisciplinarity), but fundamentally change the involved disciplines in some respect producing transdisciplinarity (Frazzetto, 2011).

During this initial three-day meeting, our team won a competition for experimental funding. In conjunction with the ENSN, Andreas Roepstorff, now Director of the Interacting Minds Center and then, an anthropologist turned neuroscientist, offered the winners of the experimental competition an opportunity to carry out their experiment without having to seek additional funding. This created a less constrictive setting in which we could think transformatively about experimental paradigms-outside the confines of normal funding structures bounded by disciplinarity. This, we admit, is a unique and enviable position in many respects-a situation that is not likely to occur again without an opening of disciplinary boundaries and institutional purses. However, and as we will detail in a moment, while the ENSN experimental competition represented the opportunity to find access to equipment, training, and the sense that humanists and social scientists could be important-if not equal-players in the experimental process, the practical reality of collaboration raised more questions than it answered (See our related paper – Fitzgerald et al., 2014 - for a fuller discussion of this event, and of the experiment that was designed).

During the subsequent five years, our research group (made up of social scientists, a neuroscientist, and a humanist) designed and executed an fMRI experiment that was intended to challenge current lie detection paradigms by inverting assumptions about “truth” as a baseline for human cognition. On its surface, our experiment questioned extant fMRI studies that define lying as the suppression of a known truth (Spence et al., 2001, 2004; Langleben et al., 2002, 2005; Ganis et al., 2003; Mohamed et al., 2006). Such studies do not situate lying in its often variable and dynamic social contexts, nor do they address the social and ethical implications of characterizing truth-telling as more biologically efficient. As a challenge to these studies, we hypothesized that “truth” can be a multi-faceted variable; that some forms of truth-telling may require elevated activation in areas of the brain associated with executive function, (decision making, planning, problem-solving and assigning priority) and theory of mind (ToM; the ability to perceive the emotional state of others and the likely social consequences of that state). In short, we hypothesized that truth may not be a suitable baseline or control variable. And, indeed, our experimental findings indicated that socially stressful truth-telling can activate portions of the brain that are associated with ToM and complex decision making (Littlefield et al., in preparation).

Yet, even as we completed what was-by many measures-a “successful” experiment, our findings led us to more deeply question the very basis of our experimental endeavor. Indeed, our transdisciplinary collaboration was also an intervention and an experiment at another level: a self-conscious enactment of, and practice in, the emergent practice of neuro-collaboration. In a related paper, we explore some of the pragmatic and affective labor involved in this enactment, particularly as it relates to how we conceive of “deception,” and how this category helps us to think more broadly about institutional rubrics of inter- and transdisciplinarity (Fitzgerald et al., 2014). In the present paper, we focus more precisely on the experience of DDC among the researchers involved.

Disciplinary double consciousness has been at work for us from our initial 2009 meeting in Vienna to our data analysis session in January 2013 and beyond; five years of living a kind of double life, having to explain ourselves to neuroscientists, humanists, and social scientists alike, and having to consistently understand and articulate ourselves through their expectations. Schematically, we break this five-year collaboration into three distinct periods during which we experienced different aspects of DDC: (1) designer, (2) data collector, and (3) analyst. The three instances of DDC we describe in this paper are exemplary moments (among many others) through which we now recognize how our own emergent DDC helped us to create, enact, and sustain this transdisciplinary experiment.

### DESIGNER OR HOW ORIGINAL CONCEPTIONS MET UNINTENDED CONSEQUENCES

The experiment on socially stressful truth was first conceived as a counter-point to current research on fMRI lie detection. If we were able to demonstrate our hypothesis (in part or in full), we hoped to challenge – but certainly not *improve* – current fMRI lie detection research. Therein lies the Hegelian rub, which we only later realized: that if we were to use the master’s tools (fMRI) to dismantle the master’s house (lie detection paradigms) we could also, potentially, aid in the production of a *better* house. But it was only by learning to experience our DDC that this double-bind became apparent. Let us examine each issue in turn: our ideal goal of upsetting current fMRI lie detection paradigms was, admittedly, somewhat naïve. As Kuhn (1970) argued, paradigm shifts are rare and must contend with the established worldview of scientific disciplines. Moreover, one experiment on the variations of “truth” would not necessarily be replicable and/or applicable to the paradigms of lie detection research that have stood for over a century. However, in our enthusiasm to create an experiment, access the tools of the neurosciences, and intervene in (what we continue to see as) a problematic paradigm, our disciplined selves did not look over or through several alternate possibilities.

It was a research participant who, in fact, taught us how to experience our DDC here. After learning the true (ideal) purpose of the experiment during a debriefing session, a male participant called back the following day to ask that his data be withdrawn from the experiment because he disagreed with current lie detection research and wanted no part in making a better lie detector. We were all taken aback: wasn’t that the exact opposite of what we were doing? None of us would have invested so many years in this experiment if it was simply going to create a better lie detector. So, we asked the participant if he would be willing to discuss his concerns with the research team. After a long discussion, in which we assured him that we were absolutely invested in (as we loftily termed it) “debunking” fMRI lie detection research, assured him that at least one member of the team (Littlefield; a humanist) had published papers and a book on the cultural history of lie detection technologies, and asked for his input on the participant side of the experiment, he enthusiastically agreed to remain part of the study.^6^ Regardless of his participation in the experiment, his participation in this post-experiment discussion was, perhaps, one of the single-most defining moments of DDC for the research team. His concerns recalled us to not only the problem at hand, but our own potential complicity within fMRI lie detection paradigms.

As (literature) science and technology studies scholars, we believed in the potential of multiplicity and critical analysis; however, we had failed to see that our participation could also be read very differently: that once the scientific paper was published it could be used in ways that were never intended at the experiment’s conception. And this was not only a question of perception-having poked at the veil of strict disciplinarity, we had very much left ourselves open to a very different system of apprehension and exchange to the one we had previously imagined. On the one hand, we still hoped to challenge fMRI lie detection paradigms; on the other, we had to confront the possibility that our position as neuroscientific experimenters was either antithetical to and/or negated the potential of our experimental ideals. Between these two, we learned that we were far from the masters of our destiny in this moment – that we had opened our work to the gaze of some other force, and one that was not always benign.

Here, and as we explore in the next examples, our sense of DDC retroactively helped us to explore and explain our conflicted positionality. DDC did not emerge immediately, but developed as our experience of working between fields continually complicated our ability to retreat to the relative stability of our home disciplines. As William James intimated, our experiences illustrated some of the ways that organized systems “can be thrown out of gear with others, so that the processes in one system give rise to one consciousness, and those of another system to another *simultaneously* existing consciousness” (James, 1890, 399). As designers, we had not anticipated the schism with which we were confronted. This does not mean it was avoidable, but, instead, that it should have been predictable. Such predictability could – and can – draw experiences of DDC to the surface, thus exposing the role of the polyglot researcher and exposing what makes her such a valuable – if troubled – experimenter.

### DATA COLLECTOR OR NOVICES TRYING ON LAB COATS

The dissonance between our hopes for the experiment and the potential reality of its reception was indeed jarring, but it was part and parcel of our position as expert-novices – in neuroscience and neuro-collaboration. Our apprenticeship began early at the Vienna Neuroschool as we took on the role of students: (quite literally) listening to lectures on physics, taking field trips to visit various scanners, and planning group presentations to be given in front of our instructors. But it was at Aarhus University while collecting data in the magnetic resonance imaging (MRI) bunker at the hospital that those of us who were not neuroscientists were faced with our limited capacity with the technologies at hand. As a motley crew of scholars embarking into undefined disciplinary territory, we keenly felt our tenuous position: we had to trust in the expertise of a radiographer, a neuroscientist, and a very large magnet. Despite our years – sometimes decades – of training in our respective disciplines, we were forced to ask: why should, and how does one maintain one’s expertise in the face of such galling inexperience? And what happens when one scholar/discipline defers to another?

For transdisciplinary neuro-collaborators, relative levels of expertise can contribute to a sense of DDC. In home disciplines, one’s expertise leads to credibility via systems of course work, examinations, credentialing, recognition by other experts, experience, and publications. In neuro-collaborations, expertise – and by extension, credibility-have yet to be defined: are the neuroscientists the experts? Are the humanists? The social scientists? How can such diverse expertise become mutually beneficial and recognized? Without external mechanisms for determining relative authority, other hierarchies begin to fill in the gaps. Take, for example, our experiences at the ENSN “Neuroschool” in Vienna. In practice, the Vienna Neuroschool of 2009 created an open learning environment that allowed for the exchange of ideas; however, its curriculum was geared toward educating humanists and social scientists about the neurosciences and not necessarily vice versa. For example, participants attended lectures on the physics of MRI and *f*MRI, visited several scanners and learned about experimental design; but they received less specific training on sociological or humanistic concepts. In other words, the school’s program was somewhat asymmetrical. Given the politics of funding and epistemic prestige in the contemporary academy, neuro-collaboration requires social scientists and humanists to go further-to not only build the necessary bridges, but cross them; neuro-collaborations are not yet about meeting in the middle, but they should be (see Fitzgerald et al., 2014, for an elaboration of this theme).

In our group, this created a default asymmetry of education and accommodation. The non-neuroscientist members of the group had to learn to speak the language of neuroscience; while our team’s neuroscientist did not necessarily need to expand his knowledge-base to include sociology or literature, science, and technology studies. When we worked at the white board, our team’s neuroscientist usually held the pen, leaving the remaining group members to play the role of students: novices whose primary role was to ask questions, learn concepts, and – ultimately –accept some normative neuroscientific knowledge if the study were to have any validity in neuroscience circles. While playing this role, our individual expertise often lay fallow while the paradigm and its components were worked out. There were specific exceptions to this position, we realized that our team had a particular advantage in its formative stage during the Vienna competition, as we lacked a neuroscientist who was trained in functional imaging. We watched as several of the other groups experienced in-fighting between the resident neuroscientists, or were slowed by self-imposed strictures concerning proper objects of disciplinary study. While our group did, eventually, have to make some concessions to “normal” neuroscientific practices in the laboratory, our initial brainstorming was relatively unbounded by disciplinary constraints and previous training.^7^ However, and overall, as Nicholas Burk notes in his study of “traditional” and “interdisciplinary” post-doctoral students, “if credibility is a discipline-specific construct (Turner, 2006; MacMynowski, 2007), then collaborators from different research communities, insofar as they represent their discipline, are put into the difficult position of deciding when to compromise their own ideals and values at the behest of another” (Burk, 2010, 12).

Our complicated positionality had everything to do with the relative weight of neuroscientific knowledge necessary for the running of an fMRI study. Methodologically, “neuro-collaboration” felt like it was amounting to “neuroscience.” This recognition played a large role in our sense of DDC as we found ourselves floating in and out of logics, expertise, and authority. Despite the length of our time together as a team, our formal training in neuroscience only amounted to about three days in Vienna. After only a month interim, our group (which now included a resident neuroscientist) was asked to present our experimental design paradigm to the MINDLab at Aarhus University. This was not an enviable position: faced with an experienced lab group of about twenty faculty, post-docs, and graduate students, our group members were not feeling particularly expert. The intent behind the presentation was not to break down the group, but to build up our study; however, the effect of the daunting task left many of us wondering if we had the ability to discuss 2x2 factorial design, pilot studies, behavioral measures, and stimulus timing. The meeting resulted in several changes to the original experimental design: we were urged to consider gender parity, choir singing as a Danish competitive activity, and the addition of in group/out group dynamics to increase the socially stressful feeling we wished to invoke. While our group certainly discussed each of these suggestions – and ultimately agreed to change the study in light of them – we were not expert enough at the time to know the impacts of each change on our study, especially given that only two of us [Tonks (clinical psychologist) and Dietz (neuroscientist)] had previously completed studies involving human subjects research.

As a team, we were aware that our position as expert-novices placed us into some risky territory: without a system of checks-and-balances. How would we know if the software for the fMRI paradigm was working correctly? Was the galvanic skin response being recorded in tandem with the stimulus? Even (something as simple as) ensuring that the data was being saved/archived correctly seemed monumentally difficult. In practice, and while we were in the hospital’s MRI bunker, the division was keenly felt. The entire team actively participated in the preparation for data collection: making sure participants followed safety procedures, providing them with prescribe stimulus before the fMRI scan, and debriefing them after their participation was completed. Notably absent, though, was our team’s collective participation in the actual scanning portion of the data collection. We observed each participant; we asked questions about the images and data that appeared on the numerous screens; we even received a lesson in setting up some of the software for the anatomical scan; ultimately, Dietz completed the scanning of each subject. Although our expertise as scholars in other fields made us keenly aware of vague global concerns (is the data being saved correctly?), we were relatively impotent when it came to translating these concerns into action. And, indeed, when we ran the experiment the first time (in Nov/Dec 2010), we received the bad news shortly thereafter: our data was useless; software for the new MRI machine had not been properly calibrated to synchronize our participants’ stimuli with their responses.

Despite this setback, the team remained intact and we re-ran the experiment a second time in Sept/Oct 2012, this time gathering usable data. During the second experimental trial we inhabited our DDC differently. Instead of accepting an expert-novice status that left several of us feeling disempowered, we re-embraced our expert status as scholars in disparate fields with a larger pool of experience from which to draw. Looking back – particularly at the pictures of our team (quite literally) trying on lab coats during a quiet moment in the bunker – it becomes clear that DDC can be used as both an excuse to defer responsibility to another expert and/or field *and* as a motivation to recall and deploy one’s own expertise. When we re-ran the experiment, the non-neuroscientists on our team took on a more active roll in checking and balancing the paradigm software construction and the data collection. While we still could not run the equipment, we learned more about running the software and initiated a more effective system of checks and balances that more effectively integrated our expert-novice position. In this case, DDC did not disappear, did not defer, but instead began to more subtly texture the process of data collection.

By recognizing the potential of neuro-collaborations to transform knowledge in the humanities and social sciences and vice versa, one cannot enter such a doubly experimental space with the assumption that one’s “home” discipline will be (or should remain) untouched by the transdisciplinary collaboration. In their essay on the current state of environmental research, Marleen Buizer, Bas Arts, and Kasper Kok have argued for the recognition of a wider-range of actors in theirs and – by extension, we would argue – other fields. “Like planning, environmental governance and associated theories of scale have witnessed shifts toward a greater involvement of a highly diverse set of “knowledge developing” actors, such as experts and practitioners, policy makers and citizens, professionals and laypersons” (Buizer et al., 2011). Buizer et al. (2011) also make the argument that transdisciplinary research groups are more self-sustaining if members remain reflexive throughout the process. They note “we ourselves could only make progress after we had explicated the truth claims that we found generally embedded in our disciplinary backgrounds and in our own positions with regard to these” (Buizer et al., 2011). However, and as Buizer and her colleagues note, the most productive collaborations involve the *recognition* of a “diverse set of “knowledge developing” actors” and *reflection* concerning the embedded truths of disciplinary knowledge claims. Each of these acts are best accomplished through collaborative efforts that enable one to highlight the strengths and weaknesses of one’s training and the disciplinary assumptions we each typically use to ground ourselves. Partnerships between neuroscientists, social scientists, and humanists not only offer a system of checks and balances, but the potential for collaborative problem solving that relies on future knowledge production.

Neuro-collaborations have the potential for the recognition and reflection outlined by Buier; they also have the potential to create new knowledge, particularly when individual actors are – at least partially – freed from the strictures of disciplinarity. This is not to say that disciplinarity is inherently constrictive, or that it would be possible to fully step outside of one’s own training. We are not suggesting that the academy should lose all boundaries and encourage underprepared researchers to run MRI magnets or engage in ethnographic work. What we are suggesting is that when scholars are asked to work outside of their home disciplines, they have the potential to inhabit other forms of consciousness, and in particular to be novices again: to ask questions, draw novel comparisons, and propose ideas that are not automatically restricted by the baggage of disciplinary training. Neuro-collaborations – like the novel collaborations highlighted in our introduction – have potential, in part, because of the unorthodox, and potentially risky, forms of consciousness they can inspire.

### ANALYST OR A BLOB OF ONE’S OWN^8^

Retrospectively, we would argue that neuro-collaboration is doubly experimental – insofar as doing neuroscience while also bringing social, cultural and humanistic targets into an experimental mode of knowledge production is *itself* a kind of experimental practice (see Roepstorff and Frith, 2012) that can help to develop DDC among the members of a transdisciplinary team. The analysis phase of our experimental endeavor necessarily meant analyzing data, but it also meant looking backward at our experiment and experience to (re)evaluate the conscious and unconscious narratives we developed to explain our positionality. We found that while neuro-collaboration involves established neuroscientific paradigms, it can also challenge them.

At the outset of the experiment we brought distinctive disciplinary consciousnesses and experimental expectations to the Vienna Neuroschool: the opportunity to interact with MRI scanners; the potential to win the experimental contest; the space to do some informal “fieldwork” among budding neuroscholars, and the very practical possibility of networking, making connections, and advancing our careers. If we are honest with ourselves, we all hoped to find *something* – maybe even make a scientific discovery – or, at the very least identify activation, ideally in exactly the “right” place. Indeed, in our pursuit of a viable experiment, it becomes easier and easier to think like neuroscientists, rather than like transdisciplinarily deployed neuro-collaborators. In the end, some of us wondered whether we were so intent on finding *something* that we lost – or, at the very least, forgot about maintaining – our critical edge. And here, again, is the problem of DDC magnified: we do not, and never have, presumed that neuroscientists are lacking a “critical edge”; we would not concede that the humanities and/or social sciences are the only disciplines with a purchase in critical, reflexive thinking; nor would we assume that in finding *something* that we had somehow betrayed our home disciplines’ skepticism and deep ambivalence about the natural sciences. And yet, all of these assumptions came to bear on our final experimental discussions and the write-up process that followed data analysis.

Within the paradigm of the ENSN, our work made sense; outside of this small transdisciplinary network and the Neuroschools that fostered our development, we were each left with the responsibility of constructing our own narrative about what kind of research we were engaged in and how it related to our home disciplines. Constructing such narratives was easier for some group members than others. Indeed, the humanist and sociologist of our group were the most overtly conflicted. For Littlefield, who lives a double life already (being jointly appointed across collages in an English Department and a Kinesiology and Community Health Department), this experiment was a reification of her daily academic existence and sense of DDC. Her fMRI experience was easy to translate to the Kinesiology Department; indeed, it brought her a measure of credibility with the scientists in the field. But with her English colleagues, Littlefield often had to justify her scientific research, which ostensibly appeared somewhat hypocritical. Had she not founded her career critiquing scientific studies of lie detection (Littlefield, 2009, 2011)? At these moments, Littlefield felt that she was defying her training and/or her predetermined academic role: she was expected to play the critical humanist, but instead she was the experimental scientist. Holding both positions concurrently required an elaborate narrative that framed the fMRI study as critical, as a new type of “neuro-collaboration.” If framed properly, this particular explanation could capture the liminal: a position that would recognize the potential for simultaneity and similarity of humanistic and scientific pursuits.

As a sociologist of the life sciences, trained within a tradition that has been (and often with good reason) hostile to the methods, assumptions and reductions of the natural sciences, Fitzgerald was among those increasingly convinced that the interpretive social sciences still needed to find some more constructive and mutual engagement with the life sciences – and especially with the emerging genomic and neurobiological sciences. This often placed him at odds not just with colleagues and friends, but with the most fundamental assumptions – for example, about the ontological primacy and explanatory power of “the social” – of his home discipline. And yet, when he worked among neuroscientists, Fitzgerald found that he did not experience any more comfort or any greater sense of familiarity – indeed, he found himself precisely inhabiting his narrow disciplinary training, worrying about when or where we would acknowledge the fundamentally social, even “constructed,” nature of the whole experience. Thus, for Fitzgerald, his DDC was not manifest in a doubled perspective, or being temporarily dislocated – but rather in a sense of being *never* at home. Among his sociological colleagues, he was attracted to the materiality and flexibility of biological accounts; among the neuroscientists, he insisted on the primacy of culture and society; this sense of betweenness as a condition-in-itself became, for Fitzgerald, the most potent experience of his own double-consciousness.

Beyond the narratives we crafted for our colleagues (and ourselves), we had to address the write-up of the experiment: what could we say – how much of the trick could we give away – and still be able to seek publication in a neuroscientific journal? While this was no Sokal Hoax (Sokal, 1996), our experimental impetus was not entirely pure. As you will recall, we planned (in our idealistic moments) to challenge an established paradigm of neuroscientific lie detection. When we did find activation in areas of the brain associated with ToM and the social brain we had some difficult decisions to make: would we add to the lie detection literature by offering up a way to improve experimental paradigms? (How) would we make the case that our experiment complicated lie detection experimentation? What community of scholars would be interested in our findings and why? Would we tell a different – more true? more accurate? – story in our Science and Technology Studies papers than in our neuroscience paper? In short, how would we craft our various narratives and for whom?

For the purposes of our arguments here, the answers to these questions matter less than the positionality of the authors. Indeed, we came to realize that many of these questions were the result of a sense of “disciplinary double-consciousness” – the experience and feeling of being trained in one discipline and deployed in another as ambassadors and interlocutors or as saboteurs and spies. As a team, we were certainly divided; as individual researchers, many of us experienced the strongest sense of DDC at this juncture. We had worked for five years to get to this point. We wanted to feel triumphant; instead, we felt conflicted. On the one hand, we felt pressure to prove that our experiment(s) worked, that our neuro-collaboration was successful: we had managed to create new neuroscientific knowledge. On the other hand, we wanted to illustrate the potential for neuro-collaboration – one that could preserve a sense of doubling and deployment. Yet, by this final stage, the social scientists and humanist among us felt somewhat deflated: if we published our results then, in some respects, our attempt at neuro-collaboration could transmogrified into, simply, neuroscience. As we tried to come to terms with these conflicted positions, we were forced, yet again, to re-consider the history of our collaboration and the resulting product.

## CONCLUSIONS: NEURO-COLLABORATION(S) AS THIRD SPACE

In his essay on the history of double consciousness, Bruce argues that one “cure” for double consciousness^9^ might be a kind of synthesis, or a blending, that did not devolve into either of its parts. To us, in all its awkwardness and optimism, this sounds like an early version of the transdisciplinarity agenda that we – and others (Frazzetto and Anker, 2009) – have been in search on behalf of a neuro-collaborations.

Du Bois’s mentor William James had speculated on the possibility of a real cure for alternating consciousness involving not the victory of one over the other but a process whereby “the dissociated systems came together,” resulting in a third, new Self, “different from the other two, but knowing their objects together.” Francis Wayland, in his earlier text, had cited a case of just such a cure of “double consciousness,” one in which a young woman’s recovery was marked by “the blending together of the knowledge acquired in [her] separate conditions.”

(Bruce, 1992, 303)

While James’ argument mitigates the political edge of what Du Bois would come to call “double consciousness,” we can find in his basic concept – as in discourses surrounding transdisciplinarity – the suggestion of a paradigm shift: “a process whereby “the dissociated systems came together,” resulting in a third, new Self, different from the other two, but knowing their objects together” (303). As of yet, the process of reaching this synthesis is unclear; however, through our experiences on a transdisciplinary research team, we have come to better understand several dimensions of DDC, as well as the potential for awkwardly, and perhaps worryingly apolitically, “blending together the knowledge acquired in [our] separate conditions” through the further development of neuro-collaborator as a live-able third position.

Importantly, we have not found a Jamesian cure for DDC; but neither, in retrospect, were we seeking such a cure. Our case study in neuro-collaboration illustrates that the process of collaborative blending necessitates the development of DDC. That – far from limiting neuro-collaborations – DDC makes possible the processes by which scholars from disparate fields can come to understand a third position, “different from the other two, but knowing their objects together.” Seeing multiple sides, understanding the potential for failure, recognizing the potential for a transformation of disciplinarity; all of these are made possible through a dissociation from one’s home discipline – a deployment – that results in and helps produce transdisciplinary perspectives. Indeed, we would argue that to find oneself “at home” in a new discipline, to be quite settled and to therefore *not* experience DDC is anathema to good neuro-collaboration as we conceive it.

Given that neuro-collaborations are an active – yet thus far largely indeterminate – area of study, we propose the following definition: neuro-collaboration results from specifically transdisciplinary (whether in practice or intent) collaborations between neuroscientists and other scholars from a variety of disciplines; these collaborations center on the central nervous system in all its rhetorical, social, and biological facticity and flexibility, in order to redefine pre-existing notions of disciplinarity and also of neuroscholarship itself, thus, simultaneous with the production and interpretation of biological measures, generating spaces for critical reflection and dialog about neuroscientific practices and representations, as well as the social and humanistic representation with which those practices (even implicitly) are invariably in dialog. Neuro-collaboration, as we define it is not about creating neurodisciplines (e.g., neuroaesthetics, neurohistory, neuroanthropology, neuroeconomics, etc), but about the theorization of their construction, harnessing of potential for mutual engagement, and opposition to their being exhausted by one-dimensional neuro-discourses. We realize that this definition is an ideal; that, in practice, neuro-collaborations rarely produce perfect partnerships between the neurosciences and other disciplines; that there are problems of access and funding, and training that need to be overcome; and that they are often cultivated within an academic landscape that actively hierarchizes different ways of producing knowledge. In particular, a wider sense of intellectual openness and generosity must be further cultivated if this way of thinking about neuroscholarship is to become at all practicable. With these caveats in mind, we propose our definition as a goal rather than a descriptor.

Because of the academy’s tendency to value the more positivistic sciences over the social sciences and humanities, partnerships between the neurosciences and other disciplines can easily turn into uni-directional monologs. Collaboration is too-often achieved through addition (“add a humanist/ethicist to the research group and stir”), rather than *transformation*; access typically means making *f*MRI hours available to a broader range of research groups instead of *reinventing* infrastructures and training opportunities; and critical reflection is often limited to reviewing representations of research (in the media, for example), instead of *rethinking* research paradigms and fundamental assumptions. In each case, transformation, reinventing and rethinking necessitate paradigm shifts, not quick fixes. The practical question, of course, is how to achieve multi-directional dialogs that will result in transformation, reinvention, and rethinking. In creating our narrative, we have attempted to represent the good and the bad, the moments of failure (Fitzgerald et al., 2014), as well as the potential for success, and – in particular – the very real problem of disciplinary double-consciousness that has left us questioning neuro-collaboration from the inside-out.

From our perspective, the emergence of initiatives that have the potential to become neuro-collaborations, such as the HBP and the BRAIN Initiative, are and will continue to be a force in disciplinary re-negotiations. Deployment across disciplinary lines creates opportunities for the members of each research group (in the form of employment, grant opportunities, etc), it also shapes colleagues’ conceptions of what is possible. Indeed, part of our hope in writing about our experimental experience in the context of neuro-collaboration is to recognize the difficulty – nay, impossibility – of the transdisciplinary partnerships of which we speak *unless* academic institutions (including funding structures, hiring lines, publications practice, and so on) are at least partially restructured in such a way as to generate more breathing-room for potentially risky collaborative scholarship.

## Conflict of Interest Statement

The authors declare that the research was conducted in the absence of any commercial or financial relationships that could be construed as a potential conflict of interest.

## Footnotes

1 See for example, http://www.theguardian.com/science/2013/oct/15/human-brain-project-henry-markram

2 See, for example, Simon Penny’s work on the ASE Program – in particular his essays “Rigorous Interdisciplinary Pedagogy: Five Years at ACE” (Penny, 2008b) and “Bridging Two Cultures: Towards an Interdisciplinary History of the Artist-Inventor and the Machine Artwork” (Penny, 2008a).

3 The CNS at ASU website maintains that “The guiding conceptual goals of CNS-ASU are two-fold: to increase *reflexivity* within nanotechnology research and to increase society’s capacity to engage in *anticipatory governance* of nanotechnology and other emerging technologies. Through this improved contextual awareness, we can help guide the path of nanotechnology knowledge and innovation toward more socially desirable outcomes and away from undesirable ones.” (http://cns.asu.edu/about). For scholarship concerning nanotechnology’s interdisciplinarity, (see Porter and Youtie, 2009a,b).

4 Other terms, such as “neuroscholar” (Johnson and Littlefield, 2011), have been suggested for these kinds of collaboration, but are, as yet, under-theorized for this kind of application.

5 Participants who were involved in the fMRI studies provided written informed consent as approved by the University of Illinois, Urbana-Champaign Institutional Review Board (#10084) and the Aarhus University Ethics Board.

6 Ultimately this participant was part of the first experimental run for which we lost all of the data-stimuli correlations; so, his data is not included in the analysis or subsequently published papers.

7 During the design phase of the experiment, Littlefield held an expert position concerning the history of lie detection research. The group was able to use this knowledge to craft a design that could potentially trouble paradigms that have been used in lie detection research for over a century. Likewise, during the design phase, and during the behavioral portion of the experiment, Tonks’ knowledge of behavioral psychology played a key roll in establishing situations that would create in-group/out-group dynamics; his expertise was also necessary for the selection of the various measures we used throughout the behavioral experiment.

8 We sometimes referred to this brain activation as “a blob of one’s own”: a phrase that captured a layman’s view of fMRI images tinged, ever so slightly with a certain deference to the potential transgression recorded by Virginia Woolf’s “A Room of One’s Own.”

9 Bruce makes it very clear – as have other scholars before him – that Du Bois himself was not in search of a “cure” for doule consciousness. The quote that follows in this paragraph is from the perspective of William James, not W. E. B. Du Bois.
